# Intravascular leiomyosarcoma of the brachiocephalic region – report of an unusual tumour localisation: case report and review of the literature

**DOI:** 10.1186/1477-7819-6-113

**Published:** 2008-10-27

**Authors:** Daniel-Johannes Tilkorn, Marcus Lehnhardt, Jörg Hauser, Adrien Daigeler, Detlev Hebebrand, Thomas Mentzel, Hans Ulrich Steinau, Cornelius Kuhnen

**Affiliations:** 1 Department of Plastic Surgery, Burn Center, Hand Center, Sarcoma Reference Center, BG-University-Hospital "Bergmannsheil", Ruhr-University Bochum, Germany; 2 Department of Plastic – Reconstructive and Hand Surgery, Diakonie Hospital Rotenburg/Wümme, Germany; 3 Dermatohistopathologische Gemeinschaftspraxis Friedrichshafen, Germany; 4 Institute of Pathology, BG-University-Hospital "Bergmannsheil", Ruhr-University, Bochum, Germany

## Abstract

**Background:**

Intravascular leiomyosarcoma is a rare tumour entity originating from venous vessel structures and most frequently affecting the inferior vena cava.

**Case presentation:**

A 69-year old patient presented with a biopsy proven leiomyosarcoma of the right supraclavicular region. Tumour resection and histological assessment verified the intravascular tumour origin arising from the internal jugular vein and extending into the surrounding soft tissue.

**Conclusion:**

In the presence of a biopsy proven diagnosis of leiomyosarcoma the rare condition of an intravascular tumour origin has to be considered even without signs of venous stases. This may result in an altered surgical strategy. Microthrombembolism and pulmonary metastases may complicate the course of the disease.

## Background

In contrast to liposarcoma and NOS sarcoma (pleomorph sarcoma not otherwise specified) previously known as malignant fibrous histiocytoma (MFH leiomyosarcoma) leiomyosarcoma only account for a small proportion of malignant soft tissue tumours in adults. References in the current literature vary between 5–10% [1].

Four main locations for tumour origin of leiomyosarcoma can be distinguished: 1. Intraabdominal/retroperitoneal 2. cutaneous 3. subcutaneous and 4. vascular. The very rare intravascular growth pattern most frequently affects the retroperitoneum especially the vena cava inferior [2] amounting to 75% of intravascular leiomyosarcoma [3].

Clinical symptoms derive from tumour growth with palpable masses or intraluminal obstruction leading to signs of venous stases and thrombosis. Extracaval venous branches are rarely the primary source of vascular leiomyosarcoma and involve venous branches of the lower extremity [2].

In this report, we describe a case of a 69-year old patient with a primary intravascular leiomyosarcoma of the internal jugular and subclavian veins. Differential diagnosis, clinical and pathological criteria for diagnosis of these rare intravascular tumours will be discussed.

## Case presentation

**Figure 1:**
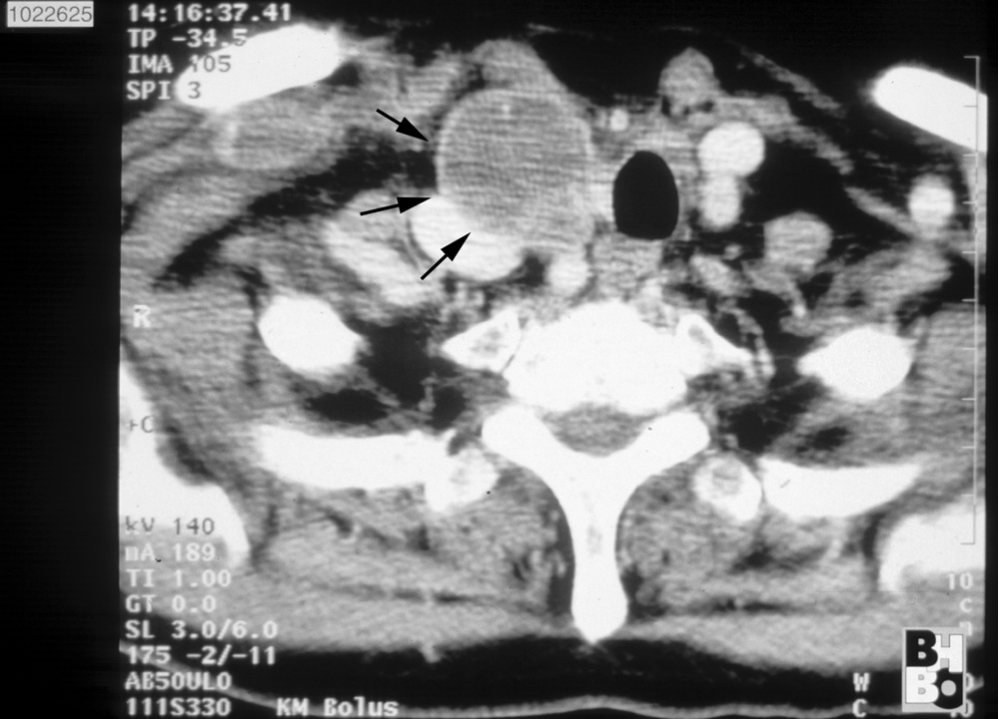
**CT scan of the neck and upper medastinum: Confirmation of a soft tissue tumour (→) 4 cm in size.** Expansive tumour growth displaced the trachea to the left and compressed the adjacent vessels.

**Figure 2:**
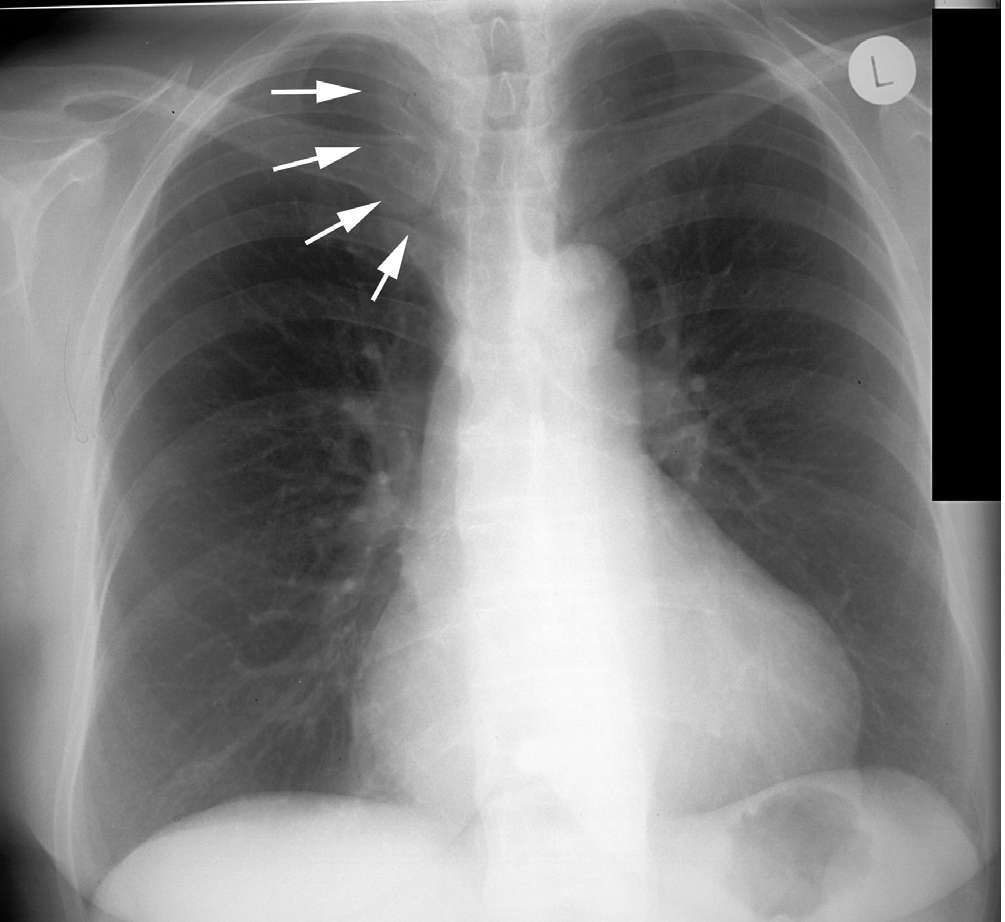
Preoperative chest x-ray displayed a mediastinal enlargement towards the right (→).

A 69-year old female patient, with a previous history of hypertension, thyroidectomy due to hyperthyroidism and hysterectomy for uterus myomas, presented with a progressive swelling of the dorsal aspect of the right side of her neck without signs of vascular obstruction or venous stases. No abnormalities of neural status of the head and neck were observed. There was no functional or sensory loss of the right upper extremity. No signs of Horner's syndrome, dysphagia, cough or dyspnoe were evident. CT scan demonstrated a retroclavicular soft tissue tumour with a cranio-caudal extension of up to 4.5 cm which partially displaced the trachea to the left and compressed the subclavian vein. An adjacent tumour of dimensions 3.5 × 3.5 cm not clearly separated from the before mentioned tumour was located at the inferior right thyroid lobe, compressing the internal jugular vein. Near the confluence of these vessels a subtotal occlusion of the brachiocephalic vein is revealed (Fig. 1). The MRI scan added no further information on the origin of the tumour or the cause of venous occlusion. There were no clear signs of tumour infiltration of the brachial plexus, brachial artery, esophagus or trachea. The preoperative chest x-ray displayed a right sided upper mediastinal enlargement (Fig. 2). Additional venous angiography indicated a filiform stenosis of the subclavian vein. Within the brachicephalic vein a longitudinal, irregular partial displacement of the vascular lumen was depicted. Extensive blood flow in cervical and supraclavicular collateral vessels was present. Neither MRI, CT nor angiogram allowed for clear distinction of the intravascular process whether it was caused by intravascular tumour growth or thrombosis. Incisional biopsy one month prior to the oncological tumour resection revealed the histopathological diagnosis of a leiomyosarcoma.

### Intraoperative findings

Surgical exposure was obtained via a triangular incision running from behind the right ear, along the anterior axillary line and across the sternum. First, the brachial plexus was dissected, the phrenic and recurrent nerves identified and followed distally. The upper border of the tumour became visible at the upper thoracic aperture. The recurrent nerve was observed to run through the tumour capsule. Further preparation was carried out from the distal edge of the wound. The pectoralis major muscle was elevated and care was taken to preserve the vascular pedicle (thoracoacromial A.V.). It was further observed that the first intercostal space was invaded by the tumour. Subsequently a thoracic wall resection including a partial resection of the right clavicle, the right half of the sternum and the costal attachment of the first three ribs was performed uncovering the mediastinum. The vena cava was revealed and trachea dissected. In this area the tumour was in close proximity to the trachea displacing it to the left but without tracheal infiltration. Next, the carotic artery and the jugular vein were exposed.

**Figure 3:**
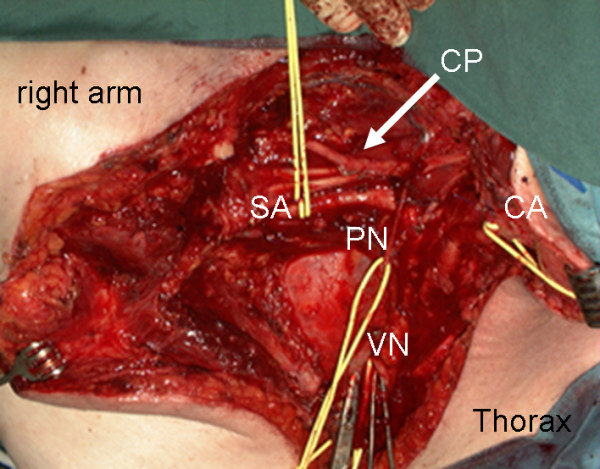
**Surgical situs: a vessel loop was placed around the subclavian artery (SA), the carotic artery (CA), the right vagus nerve (VN) and the phrenic nerve (PN).** CP indicates the cervical plexus; Clamps were placed on the stumps of the cut superior vena cava. The retractor on the left edge held back the pectoralis major muscle, in the center the exposed lung apex is visible.

The tumour, located in the right supraclavicular region/upper mediastinum, was found to surround both the subclavian and the internal and external jugular vein. Hence a resection of the subclavian vein proximal to its conjunction with the superior vena cava was required. The internal as well as the external jugular vein were incorporated into the tumour conglomerate (Fig. 3). The tumour was resected en bloc. A partial resection of the clavicle, partial resection of the sternum with removal of the brachiocephalic, sublcavian and right jugular vein and the recurrent nerve was necessary to obtain clear resection margins. The defect coverage was achieved by a pedicled myocutaneous pectoralis major island flap.

### Macroscopic and microscopic appearance

Within the surgical specimen multiple nodular polypoid tumour masses of soft consistence with diameters of up to 3.6 cm, immediately adjacent to vascular structures of the subclavian, internal jugular and brachiocephalic vein were present. The tumour with its intravascular and extravascular components comprised a total area of 7.6 × 8 × 3.3 cm. The largest intravascular tumour sprout extended close to the resection surface of the vessel.

The macroscopic appearance resembled an intravascular tumour originating from the subclavian vein with infiltration of extravascular structures.

**Figure 4:**
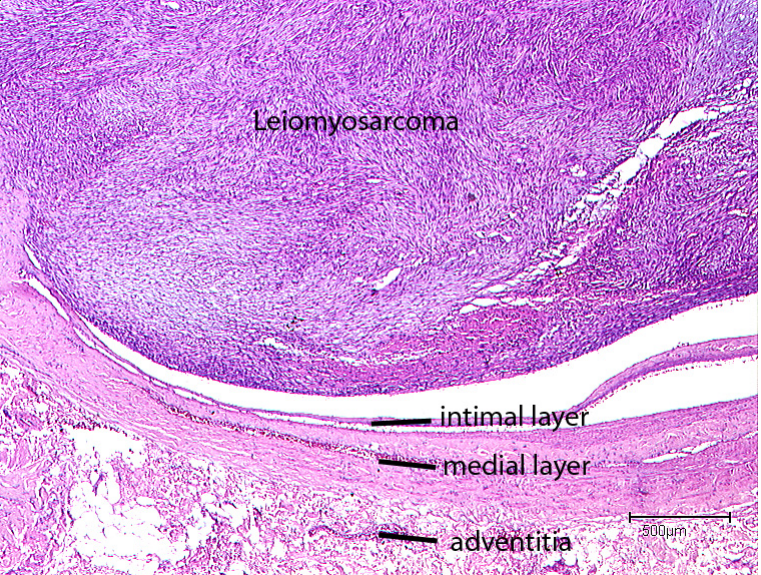
Intraluminal tumour growth of a Leiomyosarcoma originating from the subclavian vein (H&E-staining).

**Figure 5:**
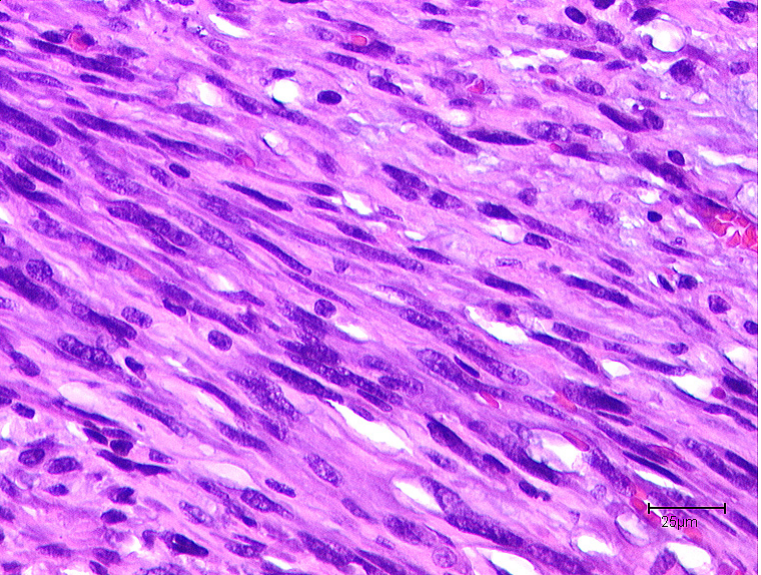
"**Cigar shaped" configurations of tumor cell nuclei of a leiomyosarcoma with nuclear atypia (H&E staining).**

Microscopically the spindle-shaped cells of this mesenchymal neoplasm originated from the media of the venous vessel wall (Fig. 4). The tumour cells formed various fascicles interwoven with other longitudinal cross sectional neighbouring fascicles (Fig. 5). The tumour cells were characterized by an eosinophilic cytoplasm and cigar shaped nuclei. The mitotic rate was 19/10 HPF (per high power field). Some foci of tumour necrosis were present.

The neoplasm derived from the media of the vessel wall, disrupted the existing vascular architecture and formed an intravascular tumour sprout.

Immunohistochemically the majority of tumour cells were positive for smooth muscle actin and desmin. A positive reaction for the proliferation marker Ki 67 was found in 25% of all tumour cells,

Thus confirming the diagnosis of an intravascular leiomyosarcoma (malignancy grading GII)

### Follow up

Postoperatively only mild signs of mixed venous and lymphatic stases of the upper extremity following the resection of the subclavian vein were observed due to the well established collateral blood flow (as seen in the preoperative angiogram). These symptoms could be positively influenced by elastic compression dressings and physical lymph drainage. Owing to the resection of the right recurrent nerve, right sided vocal cord palsy occurred. Logopaedic training was initiated. The patient recovered well and was discharged two weeks later. Both pre- and post-operatively no symptoms of pulmonary embolism were detected.

Unfortunately the patient declined the recommended radiation therapy.

After an initial 5 month of tumour free survival without evident signs of either local or systemic metastasis a tumour relapse was detected. At this stage the patient refused further treatment apart from a palliative chemotherapy.

## Discussion

Vascular leiomyosarcoma represent only a small proportion of soft tissue leiomyosarcoma [2]. These rare tumours mainly derive from structures of venous vessel walls [4], but single cases of arterial origin have been reported. With 75% of cases the inferior vena cava was identified as the main source for these intravascular tumours [3]. Venous obstruction and a palpable abdominal mass are common symptoms. Occasionally, the symptoms of the intravascular tumour growth can mimic symptoms of venous thrombosis [5].

Leiomyosarcoma deriving form smaller vessels are an exception which may lead to nervous or arterial compression due to increased pressure within the neurovascular sheets[2]. These tumours often protrude through small lumina of adjacent venous branches [6].

In the patient collective of the plastic surgery department at the University of Bochum out of the 90 soft tissue leiomyosarcoma 8 cases presented with a clear vascular origin of the tumour. In the above described case, the tumour was localized in the internal jugular and subclavian vein, in the remaining 8 cases the tumours were found in the femoral vein.

In the current literature unusual manifestations of intravascular leiomyosarcoma were described for venous branches of the lower extremity [7] whereas only single case reports of tumour manifestation of the upper extremity, the head and neck region and azygos vein [8] were found [3,9,10].

A study of 42 patients with leiomyosarcoma of the deep somatic soft tissue indicates that the predominant source of these rare malignant tumours are the small venous structures [11].

### Diagnosis of intravascular tumours

The clinical picture of an upper venous stasis may be caused by a number of different malignancies such as lung cancer and lymphomas [12]. In particular, intravascular neoplasm may lead to stasis of the blood flow through intraluminal obstruction [13,14]. Preoperative angiograms with the according filling defects, CT scans and MRI in conjunction with the clinical signs of vascular compression are useful tools in the diagnostic and operative planning of intravascular leiomyosarcoma. MRI scan can assist in differentiating an intravascular tumour growth form thrombosis. The former is represented as an homogenous tumour with an intermediate signal intensity on T1 – weighted imaging whereas a thrombus is of high signal intensity on T1 and T2 sequences [15]. The presented case underlines the difficulty in the preoperative interpretation of the origin of an intravascular leiomyosarcoma with unusual localization and tumour progression extending over the normal vascular structures into the surrounding soft tissue lacking the clinical picture of venous stases.

Moreover CT scan and MRI will not in all cases allow for an exact image of the endovascular tumour component [14] as in this particular caseTumours of the venous vessel wall may present with an intraluminal growth pattern or may extent from the tunica media and infiltrate the surrounding soft tissue [9]. Especially in thin veins extension into the perivascular soft tissue may occur early [2]. Since vascular leiomyosarcoma are often composed of an intraluminal as well as extravascular tumour component [6] on biopsy diagnosis of a soft tissue leiomyosarcoma it is necessary to consider the rare possibility of a primary intravascular tumour growth which may influence the surgical strategy. Primary intravascular tumour growth may require careful preparation and resection of the venous course affected by the malignancy.

Such tumour localizations result in both pre- and postoperative pulmonary microthrombembolism as frequent complications particularly in tumours of the pulmonary artery [16].

Furthermore, pulmonary metastases as the preferred distant tumour manifestation must be considered in the oncological care and staging [11].

### Differential diagnosis

As a malignant mesenchymal tumour, leiomyosarcoma displays differentiation tendencies towards smooth muscle morphology. Hence, histologically spindle shaped cells with eosinophilic cytoplasm with muscular striation and cigar shaped rounded nuclei can be observed. The cytoplasm is rich in contractile fibers (proteins) such as actin, desmin as well as h-caldesmon.

The differential diagnosis includes the spectrum of spindle cell shaped neoplasm. Mesenchymal tumours, the benign and malignant tumours of the nerve sheaths myofibrolastic tumours (myofibromatosis, fibromatosis, myofibroblastic sarcoma), synovial sarcoma, fibrosarcoma and NOS (not otherwise specified) sarcoma have to be considered [17].

In addition to histomorphology using standard H&E staining immunohistochemical staining for smooth muscle markers facilitates the correct diagnosis. The intravascular leiomyomatosis is characterized by the proliferation of smooth muscle vascular structures of the uterus or its surrounding. Intimal sarcoma, malignant mesenchymal tumours of the large arteries which originate from the intimal layer of the vessel wall and present as fibroblastic or undifferentiated sarcoma [18] and the very rare intravascular angiosarcoma belong to the differential diagnosis of malignant intravascular tumours [19].

### Therapy and prognosis

Complete surgical resection of the vessel segment is the therapy of choice. When an intravascular tumour origin is suspected, a ligation of the vessel far distant from the palpable tumour mass might be necessary due to considerable expansion of the intraluminal tumour sprouts [6,10].

Leiomyosarcomas of a vascular origin appear to be associated with a more aggressive tumour growth and poorer prognosis compared to respective tumours of the soft tissue [7]. Incomplete tumour resection requires adjuvant radiation therapy. Tumour size and localization are of prognostic value [9].

Leiomyosarcomas of the inferior vena cava appear to have no adverse prognosis compared to other tumour localizations [20].

The intravascular growth of the sarcoma predisposes for hematogenic metastases [11]. Hence pulmonary metastasis has to be considered in the oncological follow up.

## Conclusion

In this report, we have presented a rare case of intravascular leiomyosarcoma in the uncommon anatomical site of the upper extremity. Such diagnosis requires a complete tumour resection as the main treatment strategy, however such approach may not be fully effective due to difficulties associated with achieving clear resection margins. The intraluminal expansion of the tumour sprout may be considerable requiring vascular grafting to bridge longer vessel segments.

The occurrence of malignant intravascular tumours may present as venous obstruction and mimic the symptoms of venous thrombosis. However, in the absence of venous stases in the rare instance of a leiomyosarcoma and close proximity to vessel structures, a rare event of an intravascular tumour origin must be considered.

## Consent

Written informed consent was obtained from the patient for publication of this case report and any accompanying images.

## Competing interests

The authors declare that they have no competing interests.

## Authors' contributions

DT conceptualized the case report, gathered the data and wrote the manuscript. ML drafted and revised the manuscript. JH gathered the clinical data and assisted with postoperative care of the patient. AD reviewed the literature. DH performed the initial surgery and took responsibility for the patient's care. MT assessed the histological specimens. HS conceptualized and supervised the process of data gathering and revised the final. CK assessed the histological specimens, aided drafting and manuscript revision. All authors read and approved the final manuscript.
